# A Highly Contiguous Genome Assembly of a Polyphagous Predatory Mite *Stratiolaelaps scimitus* (Womersley) (Acari: Laelapidae)

**DOI:** 10.1093/gbe/evab011

**Published:** 2021-02-02

**Authors:** Yi Yan, Na Zhang, Chenglin Liu, Xinran Wu, Kai Liu, Zhan Yin, Xuguo Zhou, Lixia Xie

**Affiliations:** 1 Department of Entomology, College of Plant Protection, Shandong Agricultural University, Shandong Provincial Key Laboratory for Biology of Vegetable Diseases and Insect Pests, Taian, China; 2 Department of Entomology, Agricultural Science Center North, University of Kentucky, Lexington, Kentucky, USA; 3 College of Life Sciences, Hebei University, Baoding, Hebei, China

**Keywords:** Laelapidae, genome annotation, comparative genomics, gene family evolution, detoxification

## Abstract

As a polyphagous soil-dwelling predatory mite, *Stratiolaelaps scimitus* (Womersley) (Acari: Laelapidae), formerly known as *Stratiolaelaps miles* (Berlese), is native to the Northern hemisphere and preys on soil invertebrates, including fungus gnats, springtails, thrips nymphs, nematodes, and other species of mites. Already mass-produced and commercialized in North America, Europe, Oceania and China, *S. scimitus* will highly likely be introduced to other countries and regions as a biocontrol agent against edaphic pests in the near future. The introduction, however, can lead to unexpected genetic changes within populations of biological control agents, which might decrease the efficacy of pest management or increase the risks to local environments. To better understand the genetic basis of its biology and behavior, we sequenced and assembled the draft genome of *S. scimitus* using the PacBio Sequel platform II. We generated ∼150× (64.81 Gb) PacBio long reads with an average read length of 12.60 kb. Reads longer than 5 kb were assembled into contigs, resulting in the final assembly of 158 contigs with an N50 length of 7.66 Mb, and captured 93.1% of the BUSCO (Benchmarking Universal Single-Copy Orthologs) gene set (*n* *=* 1,066). We identified 16.39% (69.91 Mb) repetitive elements, 1,686 noncoding RNAs, and 13,305 protein-coding genes, which represented 95.8% BUSCO completeness. Combining analyses of genome family evolution and function enrichment of gene ontology and pathway, a total of 135 families experienced significant expansions, which were mainly involved in digestion, detoxification, immunity, and venom. Major expansions of the detoxification enzymes, that is, P450s and carboxylesterases, suggest a possible genetic mechanism underlying polyphagy and ecological adaptions. Our high-quality genome assembly and annotation provide new insights on the evolutionary biology, soil ecology, and biological control for predaceous mites.

**Significance**

Recently, *Stratiolaelaps scimitus* has been revealed as a potential biocontrol agent against edaphic pests of agricultural importance. To date, 32 Acari genomes, including 21 mites and 11 ticks have been public. A total of 135 families experienced significant expansions, which were mainly involved in digestion, detoxification, immunity, and venom. Major expansions of the detoxification enzymes, that is, P450s and carboxylesterases, suggest a possible genetic mechanism underlying polyphagy and ecological adaptions. Our high-quality genome assembly and annotation provide new insights on the evolutionary biology, soil ecology, and biological control for predaceous mites.

## Introduction

The family Laelapidae comprises a multitude of morphologically and behaviorally diverse mesostigmatic mites that are free living or associated with arthropods, mammals, or birds (Lindquist et al. 2009). Some laelapid mites, especially the subfamily Hypoaspidinae, show important potential for use as biological control agents against agricultural pests, such as *Stratiolaelaps scimitus* (Womersley). This species which often appears to have been confused with *S. miles* (Berlese) and has been distinguished from the later based on comparisons of mitochondrial DNA sequences and morphological analysis (Walter and Campbell 2003; Yan et al. 2020). In nature, *S. scimitus* is broadly distributed throughout the Holarctic, and with it is widely marketed for use in mushroom house and greenhouse production systems to manage pests (Walter and Campbell 2003; Xie et al. 2018). Additionally, *S*. *scimitus* can prey on some pests, including soil-pupating western flower thrip (Thysanoptera: Thripidae) in greenhouses, springtails (Collembola) and fungus gnats (Diptera: Sciaridae) in mushroom production facilities, and bulb mites (Acari: Astigmata) on lilies (Cabrera et al. 2005). Recently, *S. scimitus* has been studied for the potential as a biocontrol agent against other edaphic pests, such as *Bradysia odoriphaga* Yang and Zhang (Diptera: Sciaridae) (Xie et al. 2018; Zhou et al. 2018). This revealed the potential for *S. scimitus* to be used against soil-inhabiting pest of agricultural importance. High-quality genomes could facilitate studies of biology, evolutionary biology, and molecular mechanisms in adaptions to environmental changes. To date, 32 Acari genomes, including 21 mites and 11 ticks (Ixodidae), have been public (NCBI, accessed December 16, 2020). Most mite genomes are of small sizes (<200 Mb) but 14 of 21 mites with poor assembly quality, that is, number of scaffolds >10,000 and N50 length <100 kb. Among them, only two predatory mite genomes can be accessed: *Galendromus occidentalis* (western predatory mite) (Hoy et al. 2016) and *Dinothrom biumtinctorium* (Dong et al. 2018; Zhang et al. 2019). Here, we present a de novo genome assembly of *S. scimitus* using Pacific Bioscience (PacBio) single-molecule real-time long reads, annotate the repeats, protein-coding genes, and noncoding RNAs (ncRNAs), and compare gene family evolution across the main Chelicerata clades, particularly those rapidly evolving families.

## Materials and Methods

### Sample Collection and Sequencing

The parthenogenetic monoisolate of *S. scimitus* used for sequencing was collected from topsoil under the bamboo of Shandong Agricultural University, Taian, Shandong, China (36.114°N, 117.064°E) in May, 2017, and was bred for more than 23 generations in our lab. A total of 100, 100, 1, 200 females were prepared for Illumina whole-genome, Illumina transcriptome, and PacBio sequencing, respectively. Genomic DNA/RNA extraction, library preparation, and sequencing were carried out at Berry Genomics (Beijing, China). Libraries were constructed with insert sizes of 20 and 350 bp, respectively, for PacBio Sequel II and Illumina NovaSeq 6000 platforms. Quality control of raw Illumina data was performed using BBTools suite v38.49 (Bushnell 2014): remove duplicates using “clumpify.sh”; trim both read sides to Q20, discard reads shorter than 15 bp or with >5 Ns, trim poly-A/G/C tails of at least 10 bp; and correct overlapping paired reads using “bbduk.sh.”

### Genome Assembly

We performed genome survey based on short-read k-mer distributions using GenomeScope v1.0.0 (Vurture et al. 2017): K-mer frequencies was estimated with 21-mers using khist.sh (one of the BBTools suite), and maximum k-mer coverage cutoffs were set as 1,000 and 5,000.

Preliminary genome was assembled using Flye v2.6 (Kolmogorov et al. 2019) with a minimum overlap between reads of 1,000 and two rounds of self-polishing (“-m 1,000 -i 2”). Redundant contigs were removed using PurgeHaplotigs v1.1.0 (Roach et al. 2018) with a cutoff of 60 for identifying a contig as a haplotig (“-a 60”). Nonredundant assembly was polished with short reads using two rounds of NextPolish v1.1.0 (Hu et al. 2020). Minimap2 v2.12 (Li 2018) was used as read aligner during all Flye and NextPolishing steps. Potential contaminant sequences were inspected with HS-BlastN (Chen et al. 2015) and Blast+ (BlastN) v2.7.1 (Camacho et al. 2009) against the NCBI nucleotide (nt) and UniVec databases. Genome completeness was assessed using Benchmarking Universal Single-Copy Orthologs (BUSCO) v3.0.2 pipeline (Waterhouse et al. 2018) against arthropod reference gene set (*n* = 1,066).

### Genome Annotation

The essential genomic elements, that is, repetitive elements, ncRNAs, and protein-coding genes, were annotated for *S. scimitus*. We masked the repeats in the genome using RepeatMasker v4.0.9 (Smit et al. 2013–2015) based on a custom library, which combined RepBase-20181026 database (Bao et al. 2015), Dfam_3.1 (Hubley et al. 2016), and a de novo species-specific library. The de novo repeat library was generated using RepeatModeler v2.0.1 (Flynn et al. 2020). ncRNAs were identified using Infernal v1.1.2 (Nawrocki and Eddy 2013) and tRNAscan-SE v2.0.6 (Chan and Lowe 2019) with only tRNAs of high confidence selected by tRNAscan-SE script “EukHighConfidenceFilter.”

Protein-coding gene models were predicted by MAKER v2.31.10 pipeline (Holt and Yandell 2011), which integrated ab initio, transcriptome- and protein homology-based evidence. Ab initio predictions, as well as gene model training, were constructed using BRAKER v2.1.5 pipeline (Hoff et al. 2016) and passed to MAKER. BRAKER trained Augustus v3.3.2 (Stanke et al. 2004) and GeneMark-ES/ET/EP 4.48_3.60_lic (Lomsadze et al. 2005) from transcriptomic data and OrthoDB protein database (Kriventseva et al. 2019) and automatically generated gene structure annotations in the genome. Input transcriptomic alignments were generated with HISAT2 v2.2.0 (Kim et al. 2019). For the transcriptomic evidence required by MAKER, we assembled transcripts with genome-guided assembler StringTie v2.1.2 (Kovaka et al. 2019). Protein sequences of *Drosophila melanogaster*, *Daphnia pulex*, *Ixodes scapularis*, *Tetranychus urticae*, *Galendromus occidentalis*, and *Varroa destructor* were downloaded from the NCBI as the protein homology information used in MAKER. Gene functions were assigned to MAKER-derived gene models using Diamond v0.9.24 (Buchfink et al. 2015) against the UniProtKB database with the sensitive mode “-more-sensitive -e 1e-5.” We also annotated protein domains, Gene Ontology (GO), and pathways (KEGG, Reactome) using InterProScan 5.41-78.0 (Finn et al. 2017) against Pfam (El-Gebali et al. 2019), Gene3D (Lewis et al. 2018), Superfamily (Wilson et al. 2009), and CDD (Marchler-Bauer et al. 2017) databases, and using eggNOG-mapper v2.0 (Huerta-Cepas et al. 2017) against the eggNOGv5.0 database (Huerta-Cepas et al. 2019).

### Gene Family Evolution

We inferred orthogroups of 11 Chelicerata species using OrthoFinder v2.3.8 (Emms and Kelly 2019) covering representative Chelicerata lineages: Merostomata (*Tachypleus tridentatus*), Scorpiones (*Centruroides sculpturatus*), Araneae (*Stegodyphus mimosarum*), Acariformes (*Tetranychus urticae*, *Dermatophagoides pteronyssinus*), and Parasitiformes (*Ixodes scapularis*, *Galendromus occidentalis*, *Tropilaelaps mercedesae*, *Varroa destructor*, *Varroa jacobsoni*, and *S. scimitus*). Most protein sequences were downloaded from the NCBI except for *T. tridentatus* (doi: 10.5061/dryad.68pk1rv). Single-copy orthologs inferred from OrthoFinder were used for phylogenetic analyses. Protein sequences were aligned using MAFFT v7.394 (Katoh and Standley 2013) with the L-INS-I strategy, trimmed using trimAl v1.4.1 (Capella-Gutiérrez et al. 2009) with the heuristic method “automated1” and concatenated using FASconCAT-G v1.04 (Kück and Longo 2014). Phylogenetic tree was estimated using IQ-TREE v2.0-rc1 (Minh et al. 2020) with the partitioning strategy (“-m MFP –mset LG –msub nuclear –rclusterf 10 -B 1000 –alrt 1000”); genes that violate models were also removed prior tree inference (“–symtest-remove-bad –symtest-pval 0.10”). Species divergence time was estimated using Markov chain Monte Carlo Tree of the PAML v4.9j package (Yang 2007). Fossil calibrations were extracted from the PBDB database (https://www.paleobiodb.org/navigator/): Scorpiones (430.5‒443.8 Ma), Arachnida (407.6‒419.2 Ma), and Chelicerata (516‒541 Ma). Gene family evolution, that is, expansions and contractions, was estimated using CAFÉ v4.2.1 (Han et al. 2013) with the single birth–death parameter lambda. Those significantly expanded families were functionally enriched (GO and KEGG) using R package clusterProfiler v3.10.1 (Yu et al. 2012) with the default significance values (*P* value as 0.01 and *q* value as 0.05).

## Results and Discussion

### Genome Assembly and Annotation

We generated 64.81 Gb (152×) PacBio long reads and 75.55 Gb (177×) Illumina short reads for assembly. The long PacBio subreads had a mean and N50 length of 12.60 kb and 15.53 kb, respectively. We estimated a genome size of 411.22‒424.59 Mb, a heterozygosity rate of 0.062‒0.068% and a repeat length of 18.27‒31.45 Mb (4.44‒7.41%) (supplementary table S1, Supplementary Material online). A single simple peak implied that the *S. scimitus* genome had a low level of heterozygosity and repetitive content (supplementary fig. S1, Supplementary Material online).

Our final draft assembly had 158 scaffolds/contigs of 426.50 Mb, a scaffold/contig N50 length of 7.66 Mb, the longest sequence of 31.29 Mb, and a Guanine-Cytosine content of 45.85%. It has the highest contiguity quality compared with five public Mesostigmata genomes. Assembly size was almost identical with the estimated ones. BUSCO assessment against arthropod set (*n* = 1,066) revealed the high completeness and very low redundancy of our assembly: 93.1% complete, 1.9% complete and duplicated, 1.5% fragmented, and 5.4% missing BUSCO genes.

We identified 16.39% (69.91 Mb) of the genome as repetitive elements. The top five abundant repeat categories were unclassified (4.73%), Long Interspersed Nuclear Elements (LINEs) (4.50%), DNA elements (2.70%), simple repeats (2.49%), and long terminal repeat (1.73%); SINE hold a very low proportion (0.06%) (supplementary table S2, Supplementary Material online). A total of 1,686 ncRNAs were identified by Infernal and tRNAscan: 257 rRNAs, 112 small nuclear RNAs (snRNAs), 16 miRNAs, 2 long ncRNAs (lncRNAs), 1 small RNA (sRNA), 1,215 tRNAs (22 isotypes), and 81 other ncRNAs (supplementary table S3, Supplementary Material online). Among 112 snRNAs, 96 were classified as spliceosomal RNAs (U1, U2, U4, U5, U6, U11), 3 minor spliceosomal RNAs (U4atac, U6atac, U12), and 10 C/D box snoRNAs; H/ACA box snoRNAs were not discovered.

MAKER pipeline predicted 13,305 protein-coding gene models. The mean lengths of genes, exons, and introns were 7,870.13, 372.35, and 1,105.66 bp, respectively. BUSCO completeness assessment using protein mode “-m prot” identified 95.8% complete, 2.3% complete and duplicated, 0.9% fragmented, and 3.3% missing BUSCO genes (table 1), implying the high completeness of our predicted gene set. BUSCO completeness against predicted genes was slightly higher than assessment against genome assembly. It indicated the Augustus-based gene prediction under genome mode within BUSCO pipeline may had weaker capability of capturing complete genes using the default fly gene model training parameters. Diamond searches aligned 11,687 (87.84%) genes to the Uniprot proteins. InterproScan and eggNOG functional annotations assigned protein domains of 10,248 (77.02%) genes; 8,943 GO terms; 7,147 KEGG ko terms; 2,576 Enzyme Codes; 4,422 KEGG and 4,083 Reactome pathways; and 9,453 COG categories, respectively.

**Table 1:** Genome Assembly and Annotation Statistics of *Stratiolaelaps scimitus*

Elements	Current Version
Genome assembly	
Assembly size (Mb)	426.50
Number of scaffolds/contigs	158
Longest scaffold/contig (Mb)	31.29
N50 scaffold/contig length (Mb)	7.66
GC (%)	45.85
Gaps (%)	0.00
BUSCO completeness (%)	93.1
Gene annotation	
Protein-coding genes	13,305
Mean protein length (aa)	500.59
Mean gene length (bp)	7,870.13
Exons per gene	6.24
Exon (%)	7.25
Mean exon length	372.35
Intron (%)	5.02
Mean intron length	1,105.66
BUSCO completeness (%) Gene families Number of orthogroups/genes Species-specific families/genes Single-copy genes	95.8 9,151/12,274 111/338 399

### Phylogeny

**Fig. 1:**
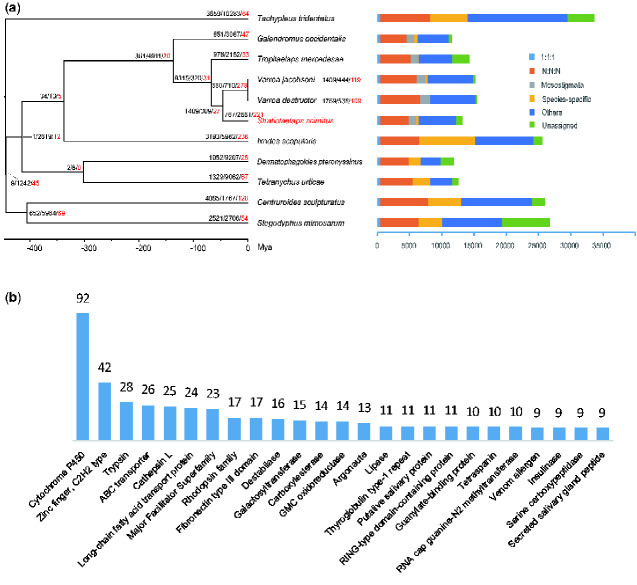
Phylogeny, orthologs, and gene family evolution. (*a*) Dating tree with node values representing the number of expanded, contracted, and rapidly evolving families. “1:1:1” represents shared single-copy genes, “N:N:N” as multicopy genes shared by all species, “Mesostigmata” as shared orthologs unique to Mesostigmata, “Others” as unclassified orthologs, “Unassigned” as orthologs which cannot be assigned into any gene families (orthogroups). (*b*) The top 25 significantly expanded families with number as the number of genes within the family. Nodes lacking bootstrap support indicated the values of SH-alrt and ultrafast bootstrap are 100/100. The *y* axis means number of genes.

A total of 89.1% (183,669) genes were clustered into 18,319 orthogroups (gene families). Among them, 3,161 families were shared by all 11species and 399 are single-copy ones; 916 families and 6,309 orthologs are common to five Mesostigmata species (fig. 1*a*). For *S. scimitus*, 12,274 (92.25%) genes were assigned into 9,296 orthogroups; 571 families and 3,672 genes were species specific.

**Fig. 2:**
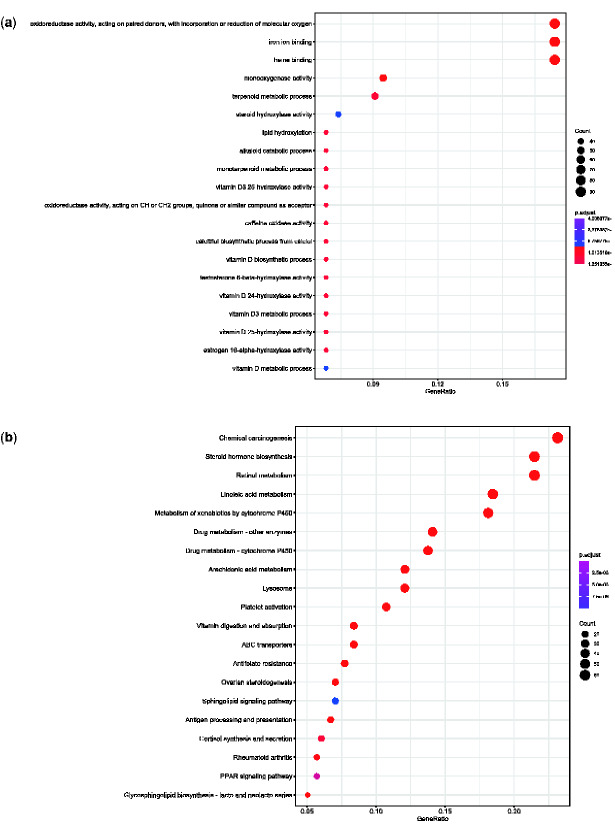
GO (*a*) and KEGG (*b*) function enrichment of significantly expanded gene families. Only the top 20 categories shown.

Thirty-nine single-copy loci were removed by IQ-TREE “symtest” prior to formal phylogenetic analyses. Phylogenetic reconstruction based on 360 single-copy loci revealed that *S. scimitus* were clustered with other four Mesostigmata species and were sistered to *Varroa* (Varroidae) rather than *Tropilaelaps mercedesae* (Laelapidae), questioning the current classification of Varroidae and Laelapidae. Considering that many members of both families acted as parasites associated with honeybees. Varroidae is possibly the ingroup of Laelapidae. Mesostigmata, Dermanyssoidea, and *S. scimitus* originated from early Cretaceous (132–142 Ma), early Paleocene (65–70 Ma), and middle Eocene (44–48 Ma), respectively (fig. 1*a*). The emergence of these Parasitiformes mites may be related to the pervasive reptiles, birds, mammals, and insects since Cretaceous.

### Gene Family Evolution

We identified 221 rapidly evolving gene families using CAFÉ, 135 and 86 of them experiencing significant expansions and contractions, respectively (fig. 1*a*). The top 25 largest expanded families were shown in figure 1*b*. Many of them are related to dietary digestion and detoxification, such as cytochrome P450, ABC transporter, carboxylesterase, trypsin, cathepsin L, long-chain fatty acid transport protein, lipase, thyroglobulin, salivary protein, and salivary gland protein. It explains the possible mechanism of the wide dietary for this predatory *S. scimitus*. The largest expanded family, cytochrome P450, obviously plays an important role in digestion and detoxification by contributing to xenobiotic metabolism, insecticide resistance, odorant, or pheromone metabolism (Feyereisen 2005). Interestingly, toxin-related proteins, that is, cysteine-rich secretory protein family referred from Pfam annotations, may be helpful for predatory progress by inhibiting both smooth muscle contraction and cyclic nt-gated ion channels (Yamazaki and Morita 2004). GO (fig. 2*a*) and KEGG (fig. 2*b*) enrichment further confirmed above hypotheses, most categories related to digestion and detoxification, such as GO terms monooxygenase activity, lipid hydroxylation, and KEGG pathways steroid hormone biosynthesis, metabolism of xenobiotics by cytochrome P450, vitamin digestion and absorption, ABC transporters, and ovarian steroidogenesis, etc. Gene family evolution provides essential evidence, supporting genetic mechanisms of polyphagy and ecological adaptions for *S. scimitus*.

## Supplementary Material

Supplementarydata are available at *Genome Biology and Evolution* online.

## Supplementary Material

evab011_Supplementary_Data
