# Supplementary material for: A Highly Contiguous Genome Assembly of a Polyphagous Predatory Mite Stratiolaelaps scimitus (Womersley) (Acari: Laelapidae)
Source: Genome Biol Evol. 2021 Feb 2;13(3):evab011. doi: 10.1093/gbe/evab011 (PMC7936031; doi:10.1093/gbe/evab011)
Supplement: evab011_Supplementary_Data [file evab011_supplementary_data.zip › Figure-S1.pdf]

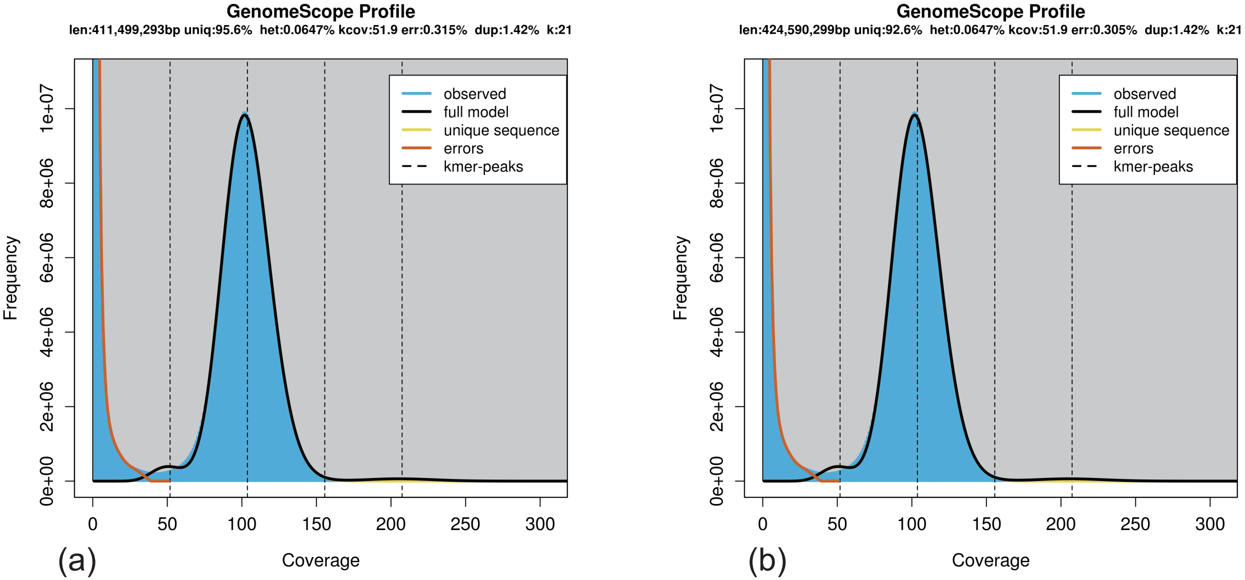

Figure S1. GenomeScope profile plots of k-mer frequency with a maximum k-mer coverage cutoff of 1,000 (a) and 5,000 (b).
